# Tube length optimization of titania nanotube array for efficient photoelectrochemical water splitting

**DOI:** 10.1038/s41598-022-27278-5

**Published:** 2023-01-03

**Authors:** Kazuki Inoue, Atsunori Matsuda, Go Kawamura

**Affiliations:** Department of Electrical and Electronic Information Engineering, Toyohashi University of Technology, 1-1 Hibarigaoka, Tempaku-cho, Toyohashi, Aichi 441-8580 Japan

**Keywords:** Chemistry, Materials science, Nanoscience and technology

## Abstract

Anodic TiO_2_ nanotube arrays (TNTAs) have attracted much attention due to their excellent photoelectrochemical (PEC) properties. In this work, the tube length of TNTAs was optimized for efficient PEC water splitting under two different conditions, in which very few or a massive amount of gas bubbles were generated on the electrodes. As a result, relatively longer TNTAs were found to be preferable for higher PEC performance when a larger number of bubbles were generated. This suggests that the mass transport in the electrolyte is assisted by the generated bubbles, so that the electrode surfaces are more easily exposed to the fresh electrolyte, leading to the higher PEC performance.

## Introduction

Water splitting for hydrogen production is an attractive solution to the current energy and environmental crisis. Especially, the hydrogen produced by solar energy is categorized as green hydrogen which is environmentally friendly because no carbon dioxide is emitted in the hydrogen production process. However, green hydrogen is still far from commercialization due to its high cost and low solar-to-hydrogen conversion efficiency, therefore related research is being conducted worldwide^1–4^.

Photoelectrochemical (PEC) water splitting is one of the promising green hydrogen production methods which can cause water splitting by applying external voltage under light irradiation. The use of porous electrode in the system is an effective way to enlarge the reaction surface area for improving hydrogen production efficiency. However, the efficiency is quite sensitive to the bubble generation especially when porous electrodes are used because, for example, the pores can be partially closed by the generated bubbles. An interesting theoretical model to investigate the influence of gas generation on the porous electrodes on the water electrolysis efficiency has been reported^5^, where only the pore size larger than 5 μm was discussed.

Anodic TiO_2_ nanotube arrays (TNTAs) with a pore size less than 100 nm are good candidate as a photocatalytic anode for PEC water splitting since they possess strong oxidative potential, large surface area and offers relatively long electron diffusion length due to their unique one-dimensional component, which is a nanotube^6^. The tube length, pore size, and wall thickness can be controlled by only changing the anodization conditions^7–11^, which is a strong advantage to optimize the structure for high efficiency of water splitting. So far, several statistic research on optimization of tube length of TNTAs for organic pollutant decomposition and photovoltaic applications has been reported^12–19^, where the optimum length for each application was varied from 1.1 to 29 μm. Table 1 lists anodization conditions, applications, and obtained and optimal tube lengths of TNTAs in previous studies. The variation of the optimum lengths among the reports was presumably due to unfixed other morphological parameters such as pore size, wall thickness, and also carbon content in the TiO_2_ wall^20^. Moreover, the optimum TNTAs tube length for gas generating application like water splitting would be different from the other cases because of, for example, the partial pore closure and light scattering by the generated bubbles; however, reliable statistic investigation was not reported till now.

**Table 1:** Reported optimum tube lengths of TNTAs for organic pollutant decomposition, photovoltaics, and water splitting.

Anodization condition			Application	Tube length (µm)		Ref
Electrolyte	Potential (V)	Duration (h)		Obtained	Optimal	
EG + NH_4_F + H_2_O	60	0.08–2	PC degradation of paraquat	1.5–25	5–7	^12^
EG + NH_4_F + H_2_O	30	1.25–3	PC degradation of MB	3.5–7	5	^13^
HF + H_2_O/CH_3_NO + NH_4_F + H_2_O	20	0.33–6	PC and PEC degradation of phenol	0.21–17	PC:12 PEC:0.21	^14^
EG + NH_4_F + H_2_O	15–60	3	PEC degradation of MO, RhB, MB	0.89–16.09	4.56	^15^
EG + NH_4_F + H_2_O	30	0.08–2	PEC water splitting	0.1–2.9	1.1	^1,0^
EG + NH_4_F + H_2_O	40	1–7	Dye sensitized solar cell	5–20	20	^16^
EG + NH_4_F + H_2_O	60	1–4	Dye sensitized solar cell	10–38	29	^17^
EG + HF + H_2_O	120	0.25–2	Dye sensitized solar cell	1–20	20	^6^
DMSO + HF + H_2_O	8–30	21–27	Dye sensitized solar cell	1.2–20	17.6	^18^

*EG* ethylene glycol, *PC* photocatalytic, *MB* methylene blue, *MO* methyl orange, *RhB* rhodamine B.

In this work, carbon-free TNTAs with fixed pore size and wall thickness were prepared to precisely investigate the effect of tube length of TNTAs on the PEC water splitting efficiency. The influence of bubble generation on the water splitting is discussed based on the experimental results obtained by changing the amount of generating gas from the TNTAs photoanodes, which provides new insights into the optimization of TNTAs morphology in water splitting application.

## Experimental

Highly ordered TNTAs were fabricated by a two-step anodization process^21^. A Ti foil was cut in 4 × 2.5 cm^2^ pieces and washed by ultrasonication in acetone for 1 h. After drying in air, the cut Ti foil as an anode and a Pt rod as a cathode were dipped into an electrolyte of ethylene glycol (EG) with 0.08 M NH_4_F and 1.3 M H_2_O, then a potential of 60 V between the electrodes was applied for 2 h at room temperature. The anodized Ti foil was ultrasonicated in deionized water for 1 h to completely remove the formed oxide layer consisting of disordered TNTAs and to obtain periodic nanovoids on the surface of the Ti foil. Second anodization was performed using the treated Ti foil as an anode and an untreated Ti foil as a cathode to obtain highly ordered TNTAs with controlled morphology. The detailed conditions for the second anodization are shown in Table 2. The samples were kept in the electrolyte at the same temperature after anodization with magnetic stirring for appropriate durations to remove the carbon-rich surface layer from TNTAs without destruction of the nanotube array structure^22^. We named this process “post anodization treatment (PAT)”. After washing with 2-propanol and drying in air, the samples were annealed at 550 °C for 1 h in air to crystallize TNTAs, followed by washing with 0.05 M HCl aqueous solution.

**Table 2:** Conditions of 2nd anodization process.

Electrolyte composition	Potential (V)	Time (min)	Temperature (°C)	PAT time (min)	Length (μm)	Pore size (nm)
0.1 M NH_4_F and 1.5 M H_2_O in 1:1 EG and DMSO (vol. ratio)	30	1	53	4	0.47	52.7
		3		5	0.95	62
		5		5	1.5	62.2
		15		6	3.7	61.1
		30		8	4.9	61.5
		60		4	9.6	80.2
0.08 M NH_4_F and 1.3 M H_2_O in EG	60	60	20	0	21	117.2

The morphology was observed with a Hitachi S-4800 scanning electron microscope (SEM, Japan). Pore sizes were measured with the ImageJ software^23^. For the tube length measurement, a part of the nanotube layer was scratched using tweezers, and the exposed cross-section was observed by tilting the sample. The crystal structure was confirmed by X-ray diffraction (XRD, SmartLab, Rigaku, Japan) and Raman spectroscopy (NRS-4500, JASCO, Japan).

The photoelectrochemical performance was evaluated by photocurrent measurement and electrochemical impedance spectroscopy (EIS) in 1 M KOH solution with a three-electrode configuration using a prepared TNTAs sample as a photoanode, a Pt coil as a counter electrode and Hg/HgO as a reference electrode (ALS Co., Ltd, Japan). Photocurrent measurements were carried out using simulated sunlight (100 mW cm^−2^, HAL-320 with an AM 1.5 filter, Asahi spectra, Japan) and UV-rays (2300 mW cm^−2^, SP-11, Ushio, Japan) as light sources, and the applied potentials were 1.23 and 1.3 V vs. RHE (reversible hydrogen electrode), respectively. The photocurrent was recorded with a VSP-300 potentiostat (Biologic, France). The applied potentials were converted to RHE scale using the equation of E_RHE_ = E_Hg/HgO_ + 0.059 pH + 0.098 V^24,25^. EIS measurements were performed under simulated sunlight irradiation (100 mW cm^−2^, HAL-320W with an AM 1.5 filter, Asahi spectra, Japan) at open circuit potential between 10 and 1 MHz with an AC amplitude of ± 5 mV using a SP-50ez potentiostat (Biologic, France). The equivalent circuit was fitted to Nyquist plots using Z view software^26^.

## Results and discussion

### Characterization of TNTAs

**Figure 1:**
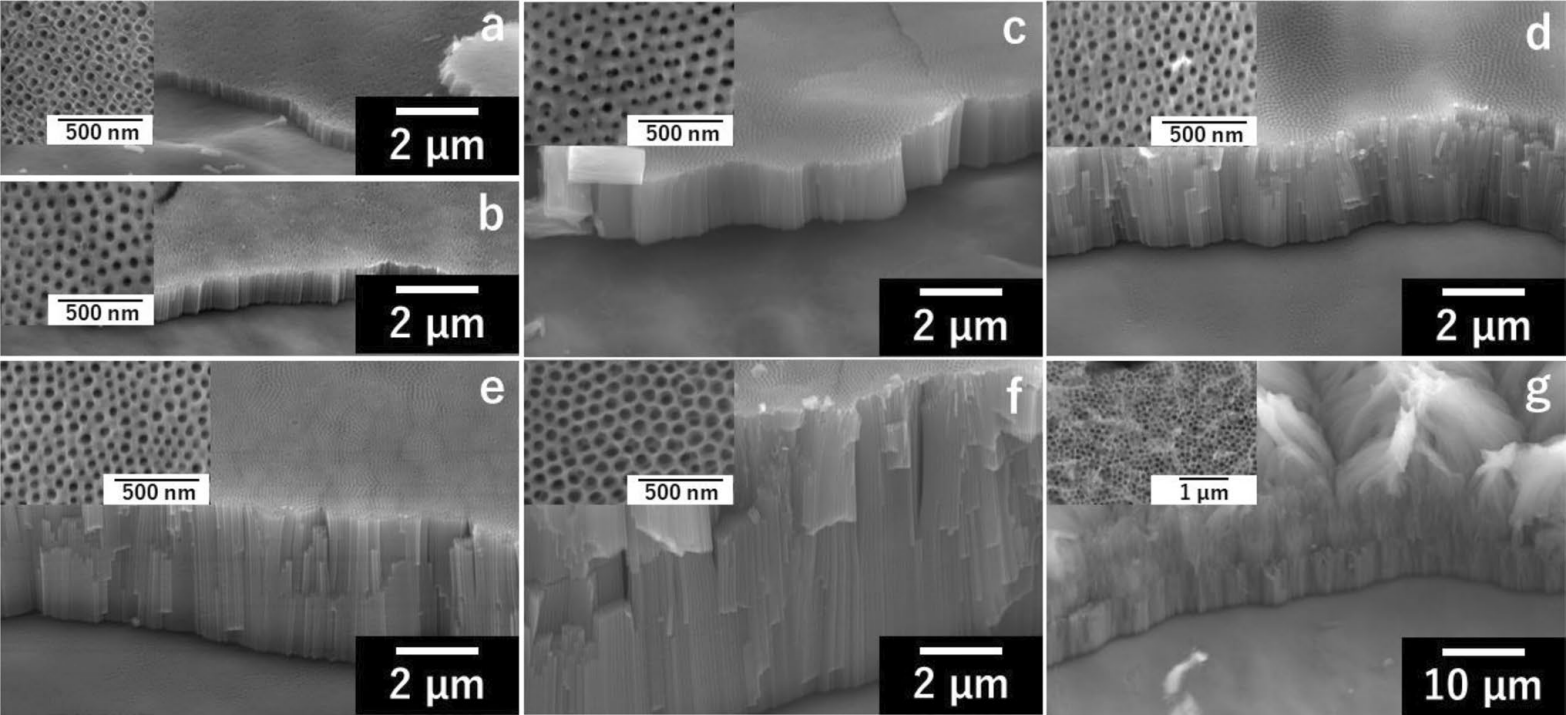
SEM images of TNTAs prepared by different anodization conditions: the voltage of 30 V was applied for (**a**) 1 min, (**b**) 3 min, (**c**) 5 min, (**d**) 15 min, (**e**) 30 min and (**f**) 60 min in an electrolyte with DMSO, and (**g**) 60 V for 60 min was applied in an electrolyte without DMSO.

Figure 1 shows the SEM images of TNTAs prepared by different anodization conditions. Highly ordered TNTAs with tube lengths of 0.47–9.6 µm were formed when 30 V was applied for different durations during the second anodization process (Fig. 1a–f). The diameter of nanotube pores was about 60 nm when the anodization time was less than 30 min and slightly increased to 80.2 nm when the time was 60 min. In general, longer anodization time leads to longer TNTAs with larger pores^9,10,12,13^. In contrast, the PAT time was controlled, for the first time, to make pore size constant in this work. This enabled to eliminate the effect of pore size on water splitting performance, thus the discussion on the effect of tube length on the performance became much simpler. On the other hand, further longer TNTAs with similar pore size were difficult to prepare, therefore the anodization conditions were drastically changed for the longest TNTAs sample in this work (Fig. 1g), resulting in a bit different morphology with bundling of nanotubes formed on top of the TNTAs. The bundle structure was formed presumably because of chemical etching by the electrolyte^27^. The tube length and the pore diameter of the longest TNTAs were 21 µm and 120 nm, respectively.

**Figure 2:**
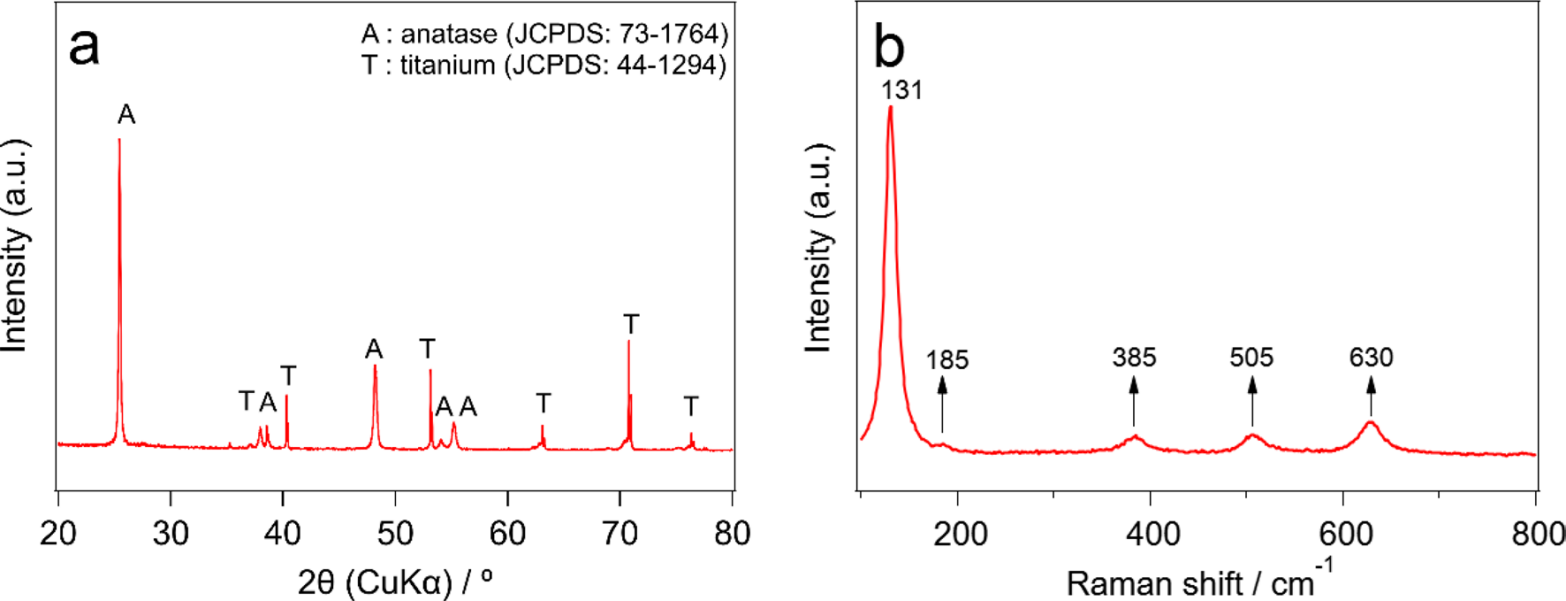
(**a**) XRD pattern and (**b**) Raman spectrum of TNTAs prepared by 1 h second anodization.

Figure 2 shows the XRD pattern and Raman scattering spectrum of TNTAs prepared by 1 h second anodization at 30 V. Diffraction peaks of metallic Ti and anatase TiO_2_ appeared in the XRD pattern^16,28^, and characteristic Raman peaks of anatase TiO_2_ were solely observed in the Raman spectrum^29–31^. These results confirmed that the prepared TNTAs were composed of a single crystal phase of anatase.

### Evaluation of photoelectrochemical properties

**Figure 3:**
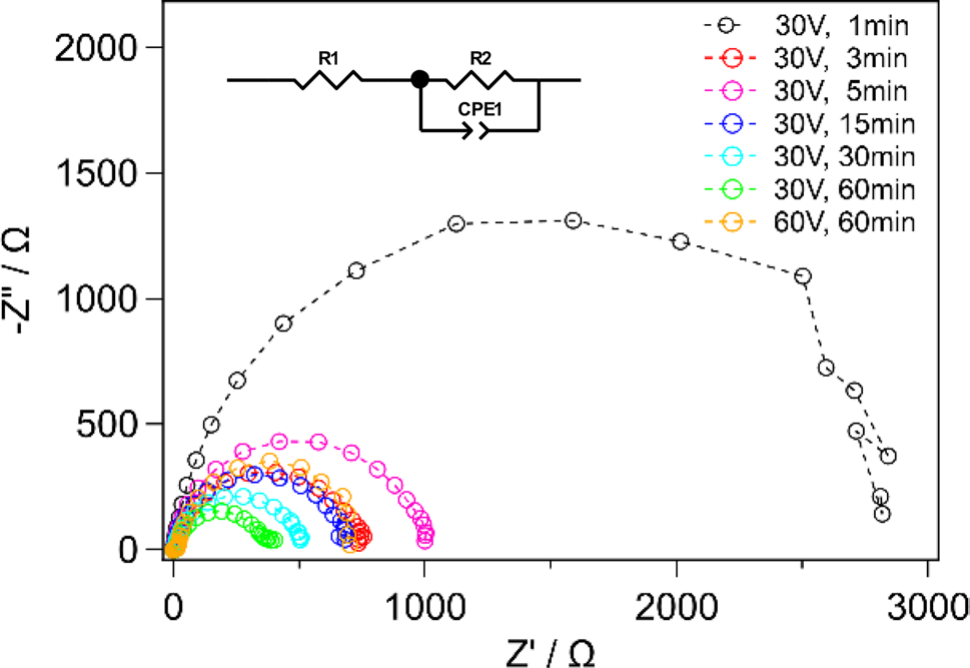
Nyquist plots of TNTAs prepared by different conditions of 2nd anodization.

Electrochemical impedance spectroscopy (EIS) was carried out to investigate the PEC properties of TNTAs as a function of the tube length. Figure 3 shows the Nyquist plots obtained from the EIS results. All the plots display single depressed semicircles, which are the characteristic of a non-homogeneous photoelectrode/electrolyte interface. Such the curves can be fitted to a simple equivalent circuit using constant phase element (CPE) as shown in the inset of Fig. 3^32^. The obtained values of circuit elements are summarized in Table 3. Series resistance, R_1_, shows a continuous increase as the tube length increases. This is simply because longer TNTAs have more chances of charge recombination in the TiO_2_ layer. On the other hand, charge transfer resistance at the interface between photoelectrode and electrolyte, R_2_, was decreased when the tube length was increased from 0.47 to 9.6 μm. This can be attributed to the fact that longer TNTAs have larger surface area where charge transportation occurs. The larger interface area also makes the TNTAs more capacitive, which is confirmed by the continuous increase in CPE values as the tube length increases. Although the PEC performance is determined by a balance among the obtained circuit elements values shown in Table 3, R2 is often most influential to the water splitting efficiency^32^. As a result, the sample with 9.6 μm would be most promising for water splitting.

**Table 3:** Parameters obtained from EIS analysis.

2nd anodization		Nyquist plot				Tube length (µm)
Potential (V)	Duration (min)	R_1_ (Ω)	R_2_ (kΩ)	CPE (µF)	n	
30	1	0.76	2.9	234	0.93	0.47
30	3	0.59	0.77	283	0.84	0.95
30	5	0.73	1.0	411	0.85	1.5
30	15	2.6	0.70	456	0.90	3.7
30	30	3.7	0.51	718	0.89	4.9
30	60	9.4	0.36	782	0.89	9.6
60	60	15	0.81	2210	0.90	21

**Figure 4:**
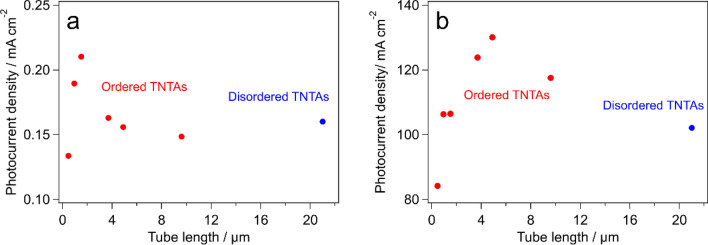
Photocurrent vs. TNTAs tube length under (**a**) simulated solar irradiation and (**b**) UV irradiation.

Figure 4a shows the photocurrent measurement result using TNTAs with various tube lengths under simulated solar irradiation. The TNTAs with 1.5 µm tube length showed the best photocurrent response. The result is roughly consistent with previous research articles reporting that too short TNTAs possess insufficient surface area, and too long TNTAs suffer from more chances of charge recombination, therefore there is an appropriate tube length between 0.21 and 29 μm (see Table 1). Figure 4b shows the photocurrent measurement result under UV irradiation. Due to the much stronger irradiation intensity, the measured photocurrent was about 350 times larger than the result in Fig. 4a. Such a large photocurrent leads to a generation of huge amount of oxygen gas on the TNTAs. In fact, a lot of bubbles were seen on the TNTAs by naked eyes under UV irradiation, whereas almost no bubbles were observed under simulated solar irradiation. Therefore, the effects of bubble generation on the photocurrent performance can be discussed by comparing Fig. 4a,b. In Fig. 4b, the largest photocurrent was achieved when TNTAs with 4.9 µm tube length was used. This is thicker than the case in Fig. 4a, suggesting that the bubble generation would facilitate mass transport in the system so that the fresh electrolyte can reach to the surface of TNTAs more easily. More specifically, the generated bubbles were rapidly detached from the surface of TNTAs because of the superhydrophilicity of anatase^33^ and moved away by buoyancy force, which causes more active convection in the electrolyte not only outside but also inside the tubular pores. The optimum tube lengths (1.5 and 4.9 μm) determined by the photocurrent measurements were a little shorter than the best tube length (9.6 μm) expected from the results of EIS. However, 4.9 µm of the optimum length obtained by the photocurrent measurement when a massive bubble was generated was closer to EIS result. This decrease in gap can be also well explained by the effect of active convection of the electrolyte by the generated bubbles because EIS measurement is carried out with alternating current supply which causes no concentration gradient in the electrolyte.

Finally, we propose future perspectives to the use of TNTAs in bubble-involved PEC water splitting. Recently, many studies for sensitization of TNTA photoanodes under visible light irradiation have been carried out, for example, by element doping, oxygen vacancy/Ti^3+^ incorporation, and plasmonic nanomaterial deposition^28,34,35^. The visible light sensitization will be related to solar hydrogen generation, thus it is important for PEC water splitting as well. However, since the introduced visible-light absorbance is generally quite weak compared to the UV-ray absorbance, very long TNTAs would be required for complete absorption of visible light from the sun light. This assumption sounds reasonable because the DSSCs need TNTAs with tube lengths longer than 20 μm (see Table 1). On the other hand, such long TNTAs with large aspect ratios suffer from diffusion suppression of electrolyte inside the nanotubes, which often limits the rate of chemical reactions. On the contrary, the results obtained in this work suggested that the large surface area of long TNTAs was efficiently used by the generated bubble-induced electrolyte convection inside and outside the nanotubes. Therefore, TNTAs photoanodes would benefit from an elongation of the tube length due to the strong visible light absorbance without suffering from diffusion suppression of electrolyte inside the nanotubes. This means TNTAs photoanodes are promising for the future use for efficient solar hydrogen generation.

## Conclusion

Highly ordered TNTAs with controlled tube lengths from 0.47 to 21 μm were synthesized by anodization process. The other morphological parameters such as pore diameter and wall thickness were fixed by controlling the PAT time, which made simple discussion on the effect of TNTAs tube length on the PEC performance possible. The XRD pattern and Raman spectrum showed the single phase anatase formation in the TNTAs. The best TNTA tube length was 1.5 μm when little amount of bubbles were formed during PEC water splitting. On the other hand, a longer tube length of 4.9 μm was the best to achieve highest efficiency of water splitting under a large quantity bubble generating condition. In addition, 9.6 μm tube length was estimated to be most efficient for PEC performance by EIS. The difference in the best tube length could be well explained from the viewpoint of the electrolyte convection inside and outside the nanotubes caused by the generated bubbles on the electrode surface, where the reaction suppressing concentration gradient was mitigated by the bubbles.

## Data Availability

The data supporting the findings of this research are available within the article. The other datasets generated during the current study are available from the corresponding author on reasonable request.
